# Neurofibromatosis Type 1 and MEK Inhibition: A Comprehensive Review with Focus on Selumetinib Therapy

**DOI:** 10.3390/jcm14145071

**Published:** 2025-07-17

**Authors:** George Imataka, Shigeko Kuwashima, Shujiro Hayashi, Kei Ogino, Eisei Hoshiyama, Katsuhiko Naruse, Hideaki Shiraishi

**Affiliations:** 1 Department of Pediatrics, Dokkyo Medical University, Tochigi 321-0293, Japan; 2 Division of Clinical Genetics, Dokkyo Medical University Hospital, Tochigi 321-0293, Japan; 3 Department of Radiology, Dokkyo Medical University, Tochigi 321-0293, Japan; 4 Department of Dermatology, Dokkyo Medical University, Tochigi 321-0293, Japan; 5 Division of Pediatric Surgery, Department of Upper Gastrointestinal Surgery, Dokkyo Medical University, Tochigi 321-0293, Japan; 6 Department of Critical Care & Medicine, Dokkyo Medical University, Tochigi 321-0293, Japan; 7 Department of Neurology, Dokkyo Medical University, Tochigi 321-0293, Japan; 8 Department of Obstetrics & Gynecology, Dokkyo Medical University, Tochigi 321-0151, Japan

**Keywords:** MEK inhibitor, neurofibromatosis type 1, selumetinib, MRI, NF1

## Abstract

Neurofibromatosis type 1 (NF1) is a genetic disorder characterized by a wide range of clinical manifestations, including café-au-lait macules, cutaneous neurofibromas, and an increased risk of certain malignancies. Historically, there has been no approved medical therapy specifically aimed at achieving tumor shrinkage or regression. Surgical intervention is often limited by factors such as the inaccessibility of the tumor location, involvement of critical tissues, suboptimal timing, or the inability to achieve complete resection. Recent advancements in targeted therapies, particularly MEK inhibitors, have introduced promising treatment options for patients with severe manifestations of NF1. This review highlights the pathophysiology of NF1 and the therapeutic role of MEK inhibitors and presents a detailed case study of a patient treated with selumetinib, a novel MEK inhibitor. While the therapeutic potential of selumetinib has been demonstrated in preclinical and clinical studies, including those involving Japanese patients, this review aims to evaluate its application in real-world clinical practice. A comprehensive discussion of the case study provides insights into the efficacy, safety, and clinical challenges associated with selumetinib treatment, offering valuable perspectives for its use in managing NF1.

## 1. Introduction

Neurofibromatosis type 1 (NF1) is a genetic tumor predisposition syndrome that affects individuals worldwide, with an estimated prevalence ranging from 1 in 3000 to 6000 people [1,2]. It is caused by mutations in the *NF1 gene* affecting nervous system growth and regulation. The condition results from insufficient neurofibromin production due to haploinsufficiency, which disrupts the regulation of the Ras–mitogen-activated protein kinase (MAPK) pathway, leading to increased cellular proliferation, overactive autocrine and paracrine signaling, and enhanced inflammatory responses in Schwann cell precursors [3,4]. It manifests as multisystem tumors, including cutaneous and subcutaneous neurofibromas, optic gliomas, and spinal or plexiform neurofibromas (PNs). PN is one of the most common tumor types in patients with *NF1*. Other features include café-au-lait spots, Lisch nodules in the iris, skeletal abnormalities like scoliosis, and vascular complications. Neurological and cognitive impairments are also common, significantly impacting quality of life (QoL) [5].

Central nervous system pathology is a major contributor to morbidity in NF1, while malignant peripheral nerve sheath tumors (MPNSTs) are the leading cause of mortality in adult patients with the condition. Imaging is essential in the diagnosis and management of NF1, playing a critical role in assessing disease progression and guiding treatment strategies [6]. A whole-body MRI (WBMRI) is recommended for detecting deep internal brain, optic, and nerve abnormalities. MRI is key for diagnosis and distinguishing benign from malignant nerve sheath tumors, with features like tumor enlargement and irregular margins suggesting malignancy [7,8]. In recent years, there has been a growing interest in targeting the MAPK pathway for the treatment of NF1-related tumors. Selumetinib (AZD6244, ARRY-142886) is an oral MEK1/2 inhibitor that effectively inhibits cell growth and induces apoptosis in tumor cells. Selumetinib has been evaluated in clinical trials involving both adults and children with various malignancies [6,9]. In April 2020, the FDA approved selumetinib (25 mg/m^2^ twice daily) for children aged ≥2 years with NF1 and inoperable PN [5]. This approval represents a significant advancement, marking the first treatment specifically designed for this condition arising from mutations in the NF1 gene. Clinical studies have demonstrated that selumetinib possesses a favorable safety profile and efficacy, particularly in achieving sustained tumor shrinkage across diverse neurofibromatosis types [6]. This sustained tumor shrinkage is observed in both benign and malignant tumors associated with different neurofibromatosis subtypes, including NF1, NF2, and schwannomatosis, highlighting the drug’s broad therapeutic potential. Selumetinib has shown consistent reductions in tumor size over time, providing long-term benefits to patients with these varied conditions. These findings underscore the potential of selumetinib as a critical therapeutic option in the management of NF1-related tumors. This review further discusses MRI use and management strategies for NF1 and presents a detailed case of a patient diagnosed with NF1 who was treated with selumetinib.

## 2. Presentation and Pathophysiology of NF1

NF1 results from a heterozygous mutation in the NF1 gene located on chromosome 17q11.2, occurring during gametogenesis. The NF1 gene encodes neurofibromin, a tumor suppressor protein that inhibits the activity of the GTPase-activating protein (GAP) domain of the intracellular signaling protein Ras (Rat sarcoma), which is involved in promoting cellular proliferation. The loss of the wild-type NF1 allele in Schwann lineage cells leads to the development of peripheral nerve sheath tumors, which is commonly associated with mutations that result in the inactivation of the remaining functional NF1 allele. However, it is important to note that NF1 is characterized by more than 3000 known germline mutations, indicating that a variety of mutations can lead to the disease. The specific mutation leading to the loss of the wild-type NF1 allele can vary, but this loss is a critical event in the development of tumors in Schwann cells. Neurofibromin’s activity is crucial across various tissue lineages, and a 50% reduction in functional neurofibromin affects all organ systems, resulting in a penetrance of 100%. This means that all patients with a pathogenic mutation will exhibit signs of the disease. Tumors typically arise after the loss of the remaining NF1 allele, particularly in Schwann cell progenitors, leading to nerve sheath tumors.

Clinically, NF1 is characterized by the presence of cutaneous neurofibromas (CNs), which originate from small nerves in the skin, and plexiform neurofibromas (PNs), which arise from Schwann cells supporting larger peripheral nerves and can involve multiple nerve bundles (nerve plexi). The diagnostic criteria for NF1 were updated in 2021 (Table 1), and patients with NF1 are at risk for both benign and malignant nerve sheath tumors, as well as neurocognitive and developmental deficits, mood disorders, osseous lesions like dystrophic scoliosis and pseudoarthrosis, and neuroendocrine tumors, including breast cancer. Early diagnosis, ideally before tumor development, is essential to offer appropriate guidance for managing potential benign and malignant neoplasms. The condition is often identified through clinical criteria (Table 1), such as six or more CALMs, neurofibromas, or Lisch nodules observed via slit-lamp examination.

The clinical manifestations of NF1 are diverse, with several types of tumors observed in the nervous system. Table 2 provides an in-depth description of these clinical manifestations and their associated tumor types. Patients with NF1 are predisposed to various non-nervous tumors and systemic complications. Hematologic malignancies such as chronic myeloid leukemia and juvenile myelomonocytic leukemia occur more frequently, with treatment similar to the general population. Breast cancer risk is significantly elevated, particularly in women under 50, with poorer prognosis due to therapy resistance; early mammographic and MRI screening is recommended. Gastrointestinal stromal tumors (GISTs) are common, often benign but symptomatic, and managed primarily through surgical resection, as they are unresponsive to imatinib. Rare pheochromocytomas and paragangliomas can cause severe symptoms like hypertension, managed through surgical resection or targeted therapies. Duodenal carcinoids are rare but distinctive, requiring surgery or somatostatin analogs. Skeletal deformities such as scoliosis, pseudoarthrosis, and osteoporosis can lead to pain and dysfunction, often requiring surgical intervention or lifestyle modifications. Pigmentation abnormalities like café-au-lait macules and Lisch nodules are common but benign, while neurocognitive deficits, including ADHD and learning disabilities, require multidisciplinary management. Patients with NF1 are prone to cardiovascular issues, including hypertension and congenital heart defects, as well as neurovascular conditions such as moyamoya syndrome and cerebral aneurysms. Management includes targeted treatments and regular monitoring to prevent complications like strokes. (https://doi.org/10.2147/JMDH.S362791 accessed on 13 June 2025).

**Figure 1:**
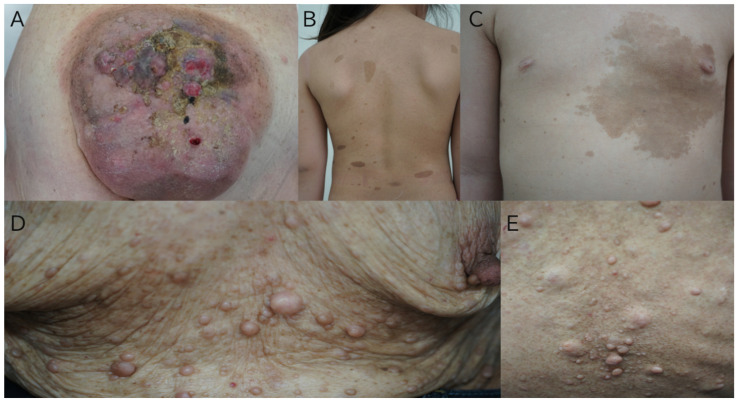
Skin lesions of NF1. (**A**) A male patient with cognitive decline. Café-au-lait macules were observed since birth. Over time, multiple neurofibromas developed, but medical attention was sought only after a large tumor in the lumbar region began to bleed. A skin biopsy confirmed the diagnosis of a malignant peripheral nerve sheath tumor (MPNST), and the patient underwent wide excision followed by post-operative radiotherapy (X-ray, total dose 50 Gy, 25 sessions). The lesion recurred afterward, and the patient passed away 18 months post-surgery without receiving additional treatment. (**B**,**C**) A 6-year-old patient with scoliosis, presenting with multiple café-au-lait macules on the back and a large café-au-lait macule on the left chest. There is no family history of neurofibromatosis. Genetic testing confirmed the diagnosis, and the patient regularly visits healthcare institutions for comprehensive treatment by various specialists for associated complications. (**D**,**E**): An elderly female patient with multiple neurofibromas on the anterior chest and back, with a history of Parkinsonism and autism spectrum disorder (ASD). She had not previously sought medical attention; nor had she been diagnosed with NF1. She was brought to the clinic for medical subsidy applications (specified diseases, rare disease certification).

The following are photos of typical skin findings of NF1 (Figure 1).

## 3. MRI in NF1

In evaluating NF1, imaging plays a crucial role in diagnosing and managing patients [10], necessitating a thorough assessment of the various affected organs to achieve accurate disease staging. This approach aids in establishing diagnostic, prognostic, and therapeutic pathways. This work aims to offer a pictorial review of neuroradiological features associated with NF1, with a focus on MRI images, outlining both technical considerations and MRI findings. Table 3 describes the MRI Characteristics and Prevalence of Common CNS Manifestations in NF1 [8]. Additionally, we analyze the prevalence of these features in a retrospective study involving a cohort of NF1 patients and compare our findings with the existing literature. MRI remains the preferred imaging method for examining brain and spinal cord lesions. It also helps differentiate between benign and malignant nerve sheath tumors, with malignancy indicators such as tumor growth, size exceeding 5 cm, poorly defined margins, absence of a central hypointense target on T2-weighted images, and heterogeneity with central necrosis [8].

Multiparametric MRI, commonly applied in prostate imaging, broadly refers to a technique that incorporates multiple sequences, such as anatomical, diffusion, and Dixon-based pre- and post-contrast imaging. Whole-body MRI (WBMRI) has been extensively investigated for its role in managing neurocutaneous syndromes, including neurofibromatosis type 1 (NF1), neurofibromatosis type 2 (NF2), and other malignancy-related conditions, due to its comprehensive imaging potential for assessing tumor burden and differentiating between various tumor types in affected patients [11].

**Figure 2:**
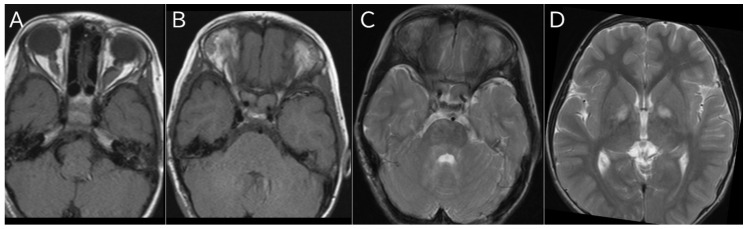
MRI imaging findings of NF1. (**A**,**B**) Bilateral optic gliomas in a 4-year-old girl; (**A**) T1-weighted image; (**B**) T2-weighted image. (**C**) T2-weighted images of UBOs in bilateral cerebellum of a 4-year-old girl. (**D**) T2-weighted image of UBOs in bilateral globus pallidus in a 7-year-old boy.

A typical MRI image of NF1 is shown (Figure 2).

## 4. Management

Physical Destruction: For individuals with numerous cutaneous neurofibromas, excision may not be practical. Carbon dioxide laser treatment under general anesthesia has been suggested for small to medium neurofibromas. However, a limitation of this method is the potential for resulting in depigmented, atrophic scars [12].

Treatment of Plexiform Neurofibromas (PNs): Treatment presents additional challenges due to nerve involvement. Surgical resection remains the gold standard treatment but is often not feasible. Alternative modalities are used for tumors that cannot be surgically removed.

## 5. Targeted Genetic Treatment

The immunosuppressant sirolimus has proven effective in slowing PN progression and reducing associated pain by inhibiting the mTOR pathway, commonly involved in NF1 tumor growth [13]. Tipifarnib, by blocking Ras signaling through inhibition of Ras farnesylation, downregulates this pro-oncogenic pathway. Imatinib, a tyrosine kinase inhibitor, has also been used for PNs, with reports of both halting tumor progression and achieving a median 26.5% reduction in tumor volume.

Direct tyrosinase inhibitors, such as kojic acid, have been proposed for targeting hyperpigmentation and café-au-lait macules (CALMs) in NF1 [14]. Other inhibitors, including MEK inhibitor PD032059 and PKA-cAMP pathway inhibitor HA1004, also target genetic pathways involved in NF1. Furthermore, given that vitamin D levels are typically lower in NF1 patients, ultraviolet B irradiation has been suggested to boost vitamin D levels and possibly reduce hyperpigmentation [15].

## 6. Role of MEK Inhibitors in NF1 and Pediatric Populations

Current MEK inhibitors (MEKi) are derived from similar chemical structures, leading to shared pharmacological characteristics. Five MEK inhibitors, all available in oral form, share generally similar metabolic and excretory profiles but differ in their half-life. They present a comparable side effect profile, commonly including rash, paronychia, reduced cardiac function, and lab abnormalities (such as elevated creatine kinase [CK] and liver dysfunction). Preclinical studies on their ability to penetrate the brain have yielded varied results, and these findings have not consistently predicted efficacy against central nervous system (CNS) tumors.

MEKi have been widely studied in adults, particularly for BRAF-mutated melanoma and other cancers. Emerging evidence also indicates that MEKi may alter the tumor immune environment, enhancing their effectiveness beyond direct tumor targeting. Although MEKi share chemical properties, they may exhibit clinically significant differences. While MEKi show promise for NF1-related conditions, no direct comparison studies of their clinical efficacy, toxicity profiles, or impact on the tumor immune microenvironment have been conducted. Currently, distinctions among MEKi in terms of formulation (particularly child-friendly options), regulatory approval, insurance availability, and NF1-specific clinical experience may be the most critical factors influencing their use [16,17,18].

## 7. MEK Inhibitors Approved for Clinical Use

Selumetinib is an oral MEK inhibitor that targets MEK1 and MEK2, key components of the Ras–RAF–MEK–ERK signaling pathway, which is frequently activated in various cancers. This inhibition prevents the phosphorylation of ERK, leading to reduced signaling that drives cell proliferation and survival. In the context of neurofibromatosis type 1 (NF1), the absence of functional neurofibromin—an essential Ras GTPase-activating protein (GAP)—results in continuous Ras activation, contributing to tumor growth. These painful and debilitating tumors are characterized by increased Ras (Rat sarcoma) and MAPK (mitogen-activated protein kinase) signaling. Treatments targeting the Ras pathway have shown limited effectiveness, as NF1 primarily arises from heterozygous loss-of-function mutations in the tumor suppressor gene NF1. According to Knudson’s two-hit hypothesis, somatic inactivation of the wild-type allele in Schwann cells of neurofibromas leads to hyperactivation of the Ras–MAPK pathway.

The NF1 gene encodes neurofibromin, which is involved in regulating Ras–MAPK signaling, and it is one of several genes associated with RASopathies. Numerous downstream pathways, including RALGDS, PI3K, RASSF, TIAM1, BRAP2, RIN, and GRB7, play roles in Ras signaling, with the Ras–MAPK pathway being the most extensively researched. In NF1, selumetinib has demonstrated promise in pediatric patients, following positive outcomes in adults with advanced cancers.

**Figure 3:**
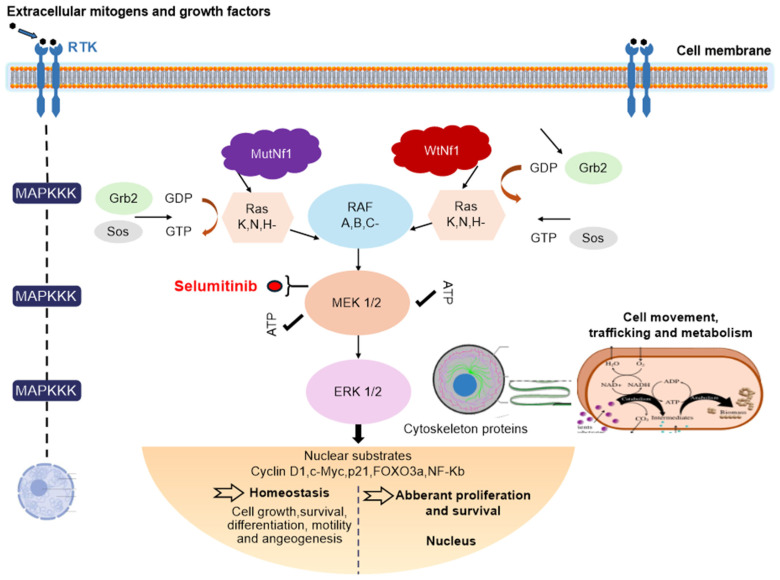
Pharmacological action selumetinib.

The MAPK pathways can be divided into three main subfamilies: JNK/SAPK (c-Jun N-terminal kinase/stress-activated protein kinase), p38 MAPK, and MEK/ERK (extracellular signal-regulated kinase). MEK contains two consensus kinase motifs responsible for phosphorylating serine/threonine and tyrosine residues. The homologues MEK1 and MEK2 share a single substrate, ERK1/2, which influences various cellular functions, including transcription, cell cycle progression, and motility. When activated, ERK, along with RAF (rapidly accelerated fibrosarcoma) and MEK, moves to the nucleus, activating cyclin D1 and downregulating p27, thus promoting cell proliferation and potentially inhibiting apoptosis through anti-apoptotic proteins (Figure 3) [6].

As upstream regulators of the ERK pathway, MEK1/2 play critical roles in many cancers. Selumetinib’s oral administration has been shown to inhibit ERK phosphorylation, resulting in decreased numbers, volume, and proliferation of neurofibromas in animal models. The NF1 gene is situated on chromosome 17q11.2 and encodes neurofibromin, a 2818-amino acid cytoplasmic protein that inhibits Ras GTPase activity by converting active GTP-bound Ras to inactive GDP-bound Ras. Dysregulation of Ras leads to heightened activation of RAF/MEK and AKT/mTOR pathways, fostering cell growth in NF1-deficient cell types.

Multiple therapeutic approaches have been investigated for NF1. Since neurofibromin acts as a Ras inhibitor, initial research focused on Ras inhibitors such as tipifarnib. Additionally, as neurofibromin regulates mTOR signaling, agents like rapamycin and its analogs are being explored for tumor treatment. Other therapies under consideration include antihistamines (like ketotifen), angiogenesis inhibitors (such as thalidomide), antifibrotic agents (like pirfenidone), and lovastatin. The absence of functional neurofibromin in NF1 patients leads to uncontrolled Ras signaling and tumor development, prompting phase II clinical trials of various agents, including tipifarnib, pirfenidone, sirolimus, pegylated interferon alfa-2b, and imatinib, with the aim of improving progression-free survival and reducing plexiform neurofibroma volume.

## 8. Pharmacological Overview of Selumetinib

MEK1/2 inhibitors were initially developed for cancer treatment in the early 2000s. Early compounds like CI-1040 did not show efficacy in lung, colon, or breast cancers. However, selumetinib, a second-generation MEK inhibitor, has proven more effective in preclinical models. The first phase I trial involved patients with advanced cancers, including melanoma, breast, and colorectal types, confirming the drug’s tolerability and safety. Subsequent phase II trials assessed its effectiveness in gastrointestinal, thyroid, non-small cell lung, ovarian cancers, melanoma, and acute myeloid leukemia. Eventually, the focus shifted from cancer therapy to treatments for rare diseases.

The recommended dosage of selumetinib is 25 mg/m^2^, administered orally twice daily on an empty stomach, available in 10 mg and 25 mg capsules. In patients with moderate hepatic impairment, the dose should be reduced to 20 mg/m^2^ twice daily. Common adverse effects, occurring in approximately 40% of patients, include vomiting, rash, abdominal pain, diarrhea, nausea, dry skin, fatigue, musculoskeletal pain, fever, acneiform rash, stomatitis, headache, paronychia, and pruritus. Serious potential side effects may include cardiomyopathy, ocular toxicity (such as retinal vein occlusion and impaired vision), and elevated creatine phosphokinase levels. As selumetinib is metabolized by the CYP3A4 enzyme, co-administration with CYP3A4 inhibitors or inducers is not advisable. Its use during pregnancy and lactation is discouraged due to insufficient data, and safety and efficacy in pediatric patients younger than 2 years have not been established. The mean oral bioavailability is around 62%, with peak concentrations reached within 1 to 1.5 h and a mean elimination half-life of 6.5 h.

Selumetinib has demonstrated considerable potential as a non-surgical treatment option for children with NF1-related plexiform neurofibromas (NF1-PNs), exhibiting the ability to reduce the size of the tumors and improve patient-reported outcomes, while maintaining an acceptable profile of tolerability [19,20,21]. The introduction of medical therapy as an alternative to surgical intervention provides additional considerations for clinical decision-making and management strategies.

In Japan, it has been approved for pediatric patients aged three years and older who have symptomatic plexiform neurofibromas associated with neurofibromatosis type 1 (NF1), particularly in cases where the tumors cannot be fully excised by surgery without significant risks of morbidity, including pain and disfigurement [22].

The approval by the Japanese Ministry of Health, Labor and Welfare (MHLW) was based on positive findings from the SPRINT Stratum 1 phase II trial, which was sponsored by the National Cancer Institute (NCI) as part of the Cancer Therapy Evaluation Program (CTEP) of the National Institutes of Health (NIH). This trial demonstrated that selumetinib, as an oral treatment, was effective in reducing the size of inoperable tumors in children [22]. Moreover, the approval was further supported by a phase I trial conducted in Japanese pediatric patients with symptomatic and inoperable plexiform neurofibromas, which also showed a reduction in tumor size.

## 9. Systematic Summary of Pivotal Clinical Trials for Selumetinib in NF1

The clinical development of selumetinib for NF1-related plexiform neurofibromas represents a landmark achievement in targeted therapy for rare diseases. The pivotal SPRINT Stratum 1 trial conducted between 2015 and 2019 established the foundation for regulatory approval by demonstrating remarkable efficacy in a pediatric population with previously limited treatment options [20]. This phase II, single-arm, open-label study enrolled 50 children aged 3–18 years (median 10.8 years) with symptomatic and inoperable plexiform neurofibromas. The primary endpoint of achieving at least 20% reduction in tumor volume by MRI at 12 months was met by 68% of patients (34/50), representing an unprecedented response rate for this condition.

The clinical benefits extended beyond tumor shrinkage, with secondary outcomes demonstrating meaningful improvements in patient quality of life. The median time to response was 5.6 months, and remarkably, 82% of responders maintained their response at 24 months [20]. Quality of life scores improved in 70% of patients, while pain reduction was observed in 85% of patients who had baseline pain, highlighting the multifaceted benefits of MEK inhibition in NF1 management. The safety profile was generally acceptable, with grade 3 adverse events occurring in 40% of patients and treatment discontinuation due to adverse events in only 6% of cases. Importantly, no treatment-related deaths were reported during the study period.

The SPRINT Stratum 2 trial further expanded the evidence base by examining selumetinib efficacy in 25 pediatric patients with asymptomatic plexiform neurofibromas at risk for developing tumor-related morbidity [21]. Despite the asymptomatic nature of these tumors, 60% of patients achieved the primary endpoint of 20% volume reduction, with a safety profile similar to the SPRINT Stratum 1 trial, and notably, no grade 4 events were reported. This finding suggests the potential utility of selumetinib in preventing progression of early-stage disease.

International validation of selumetinib efficacy was provided through a Japanese phase I dose-escalation study involving six pediatric patients with symptomatic and inoperable plexiform neurofibromas [22]. Remarkably, five of six patients (83%) achieved tumor reduction, with a median time to response of 4.2 months, slightly faster than observed in the SPRINT trials. The adverse event profile was consistent with previous studies, though skin toxicity was universal (100%) and gastrointestinal events occurred in 83% of patients. Notably, no patients discontinued treatment due to adverse events, suggesting good tolerability in the Japanese population.

Long-term follow-up data from the SPRINT trials have provided reassuring evidence of sustained efficacy and safety. The median duration of response has not been reached after 36 months of follow-up, with 88% of patients remaining on therapy at two years [20,21]. Functional outcomes demonstrated clinically meaningful improvements, with enhanced mobility observed in 45% of patients who had baseline impairment; these outcomes also demonstrated reduced disfigurement scores in 60% of patients. These findings underscore the potential for selumetinib to address both the physical and the psychosocial burdens of NF1.

Comparative analysis across international populations reveals consistent efficacy, with response rates ranging from 68% in US/European populations to 83% in the Japanese cohort, yielding a combined response rate of 69% [22]. The time to response was remarkably consistent across populations (5.4–5.6 months), while grade 3 or higher adverse events occurred in 33–40% of patients. Treatment discontinuation rates remained low across all studies (0–6%), supporting the general tolerability of selumetinib in diverse pediatric populations.

The adverse event profile of selumetinib requires careful management but is generally predictable and manageable. Gastrointestinal events, including vomiting (42%), diarrhea (36%), and abdominal pain (38%), represent the most common category of adverse events [19,20]. Dermatologic toxicities, particularly rash (40%), acneiform eruptions (28%), and paronychia (22%), are frequent but typically manageable with topical treatments and dose modifications when necessary. Elevated creatine phosphokinase levels, observed in 58% of patients with 14% experiencing grade 3 or higher elevations, require regular monitoring but rarely necessitate treatment discontinuation (Table 4).

Patient-reported outcomes provide compelling evidence of clinical meaningfulness beyond tumor measurements. Pain assessment using validated scales demonstrated a 50% reduction in mean pain scores from 4.2 at baseline to 2.1 at 12 months [20]. Quality of life improvements were substantial, with physical functioning scores increasing by 12.3 points and emotional functioning by 8.7 points on the PedsQL scale. Functional assessments revealed that activities of daily living improved in 58% of patients, while school or work attendance improved in 42% of patients, demonstrating the broad impact of effective NF1 treatment on patient functioning.

The evidence quality supporting selumetinib use in NF1 is high, based on well-designed phase II prospective studies with appropriate endpoints and statistical power [19,20,21]. Regulatory approval by the FDA and subsequently by the Japanese Ministry of Health, Labour and Welfare was based primarily on the robust SPRINT Stratum 1 results, with international validation provided by the Japanese phase I study. Long-term safety has been established through extended follow-up exceeding three years, providing confidence in the drug’s chronic use profile.

Clinical decision-making strategies regarding selumetinib therapy should consider several key factors that have emerged from the clinical trial experience. Patient selection appears optimal for those with symptomatic, inoperable plexiform neurofibromas, though emerging evidence suggests potential utility in asymptomatic patients at risk for progression [21]. Response prediction analyses suggest that younger patients and those with smaller baseline tumor volumes may be associated with better response rates, though these factors should not exclude patients from consideration given the individual variability in response. Monitoring requirements include monthly assessments for the first three months of therapy, followed by quarterly evaluations thereafter to detect both efficacy and safety signals. Treatment duration should be continuous, as the median treatment duration in responders exceeds 24 months and discontinuation typically results in tumor regrowth.

## 10. Conclusions

The introduction of MEK inhibitors, particularly selumetinib, marks a significant advancement in the management of NF1, offering a targeted treatment for a condition that was previously difficult to address. By leveraging insights into the Ras–MAPK pathway, these therapies aim to reduce tumor burden and enhance the quality of life for individuals with NF1. Nevertheless, further research is needed to optimize treatment approaches, overcome existing challenges.

As precision medicine continues to evolve, the outlook for NF1 management grows increasingly optimistic, bringing renewed hope to patients and families navigating this complex disorder.

## Figures and Tables

**Table 1:** Diagnostic criteria for NF1 (revised 2021).

Diagnostic Criteria	Clinical Context
Two or More of the Following:	
1. Café-au-lait macules (CALMs)	Presence of six or more CALMs, at least 5 mm in diameter in pre-pubertal individuals or 15 mm in post-pubertal individuals, suggests NF1.
	Having only two or three CALMs is generally normal.
	CALMs usually appear in infancy and have smooth, well-defined edges.
2. Neurofibromas	Diagnosis includes two or more neurofibromas of any kind or one plexiform neurofibroma.
	Dermal and subcutaneous neurofibromas typically develop later in childhood.
	Plexiform neurofibromas may cause changes in skin texture or color.
3. Axillary or inguinal freckling (Crowe sign)	These freckles typically develop after age 5.
	Freckles in non-sun-exposed areas are unusual in individuals without NF1
4. Optic gliomas (OPGs)	Often invisible without specialized eye exams but may be present in infancy.
	Early detection is vital to protect vision.
5. Iris hamartomas (Lisch nodules)	These age-dependent nodules are uncommon in infants and toddlers but appear in most teenagers.
	They require a slit-lamp examination for detection and do not affect vision.
6. Osseous abnormalities	Typical findings include tibial dysplasia, such as bowing, cortical thickening, or pseudoarthrosis.
	Infants or toddlers with bowing of the tibia should undergo radiography and be referred to an orthopedic specialist.
7. Family history	A first-degree relative (parent, sibling, or child) with NF1 based on diagnostic criteria confirms familial inheritance.
	NF1 is fully penetrant, meaning symptoms will always be present, even if mild, and it does not skip generations.

**Table 2:** Nervous Tumors of NF1 (https://doi.org/10.2147/JMDH.S362791 accessed on 13 June 2025).

Tumor Type	Description	Key Features
Plexiform Neurofibromas (PNs)	Occur in ~60% of NF1 patients; often congenital and arise from neural crest cells. Grow concentrically within large nerves, causing potential disfigurement and nerve dysfunction.	Unlimited growth; can lead to pain, neuropathy, or ambulation interference. Risk of malignant transformation (~15%) to Malignant Peripheral Nerve Sheath Tumors (MPNSTs).
Malignant Peripheral Nerve Sheath Tumors (MPNSTs)	Aggressive form arising from pre-existing PNs, primarily affecting individuals aged 20–40 years. Poor prognosis with a high risk of recurrence and metastasis.	Radio- and chemo-insensitive nature; gross total resection required for potential cure. 5-year survival ~50%; median survival for inoperable/metastatic cases reduced to 11–12 months.
Cutaneous Neurofibromas (CNs)	Skin tumors originating from cutaneous nerves or hair bulge cells. Form small, rubbery nodules, usually amelanotic or erythematous. Commonly appear in adolescence.	Limited growth potential; minimal risk of malignant transformation. Removal options include surgical resection, CO_2_ laser ablation, and advanced photocoagulation methods.
Subcutaneous Neurofibromas (SCNs)	Exhibit indistinct borders and appear violaceous. Typically develop during late childhood or adolescence, mostly on the trunk, arms, and face.	Cause discomfort, itching, and visible disfigurement; rarely grow larger than 2 cm. Physical removal or destruction is the only effective treatment.
Gliomas	Predominantly Optic Pathway Gliomas (OPGs), with a smaller subset of high-grade gliomas. Often occur in NF1 patients within the first 5 years of life.	OPG is benign in most cases (~75–80% 5-year survival) but can lead to vision loss or endocrine issues. High-grade gliomas have a 50-fold increased risk in NF1.
Optic Pathway Gliomas (OPGs)	WHO grade 1 astrocytic tumors often associated with NF1. Frequently observed in 15–20% of NF1 patients, manifesting early in life and sometimes spontaneously regressing.	Vision impairment and/or endocrine disturbances may occur. Treatment options include chemotherapy (e.g., carboplatin and vincristine) or MEK inhibitors; surgery is rarely used.

**Table 3:** MRI Characteristics and Prevalence of Common CNS Manifestations in NF1 [8].

Condition	% of Patients Affected	MRI Characteristics
Brain Tumor	Varies by tumor site and grade	In pediatric patients, low-grade gliomas are common, primarily affecting the cerebellum and brainstem; in adults, high-grade gliomas are more frequent, mainly in the cerebral hemispheres. On T2-weighted scans: hyperintense areas. On T1-weighted scans: isointense or slightly hypointense, with enhancement following gadolinium.
Brain Abnormalities (Unidentified Bright Objects; UBOs or FASI)	43–93%	Regions with increased signal on T2-weighted MRI, absent on T1-weighted scans, with no mass effect or enhancement; these anomalies are seldom found in individuals over 20 years old. The cerebellum, brainstem, and basal ganglia are frequently affected areas.
Plexiform Neurofibromas	Up to 30%	Most frequently located in the craniomaxillofacial area. On T2-weighted scans: heterogeneous masses with high signal and a low signal center. On T1-weighted scans: slightly hyperintense compared with muscle tissue, with varying degrees of contrast enhancement.
Spinal Tumors (Peripheral Nerve Sheath Tumors and Intramedullary Tumors)	40–96%	Primarily benign neurofibromas, while intramedullary tumors are uncommon. Neurofibromas show a high signal on T2-weighted scans with a central low signal target and a low signal on T1-weighted images, displaying varied contrast enhancement. Indicators of malignant transformation in nerve sheath tumors include increasing size, tumor diameter >5 cm, unclear margins, absence of central low signal target on T2, and central necrosis.
Lesions in the Visual Pathway (Optic Pathway Gliomas)	5–15%	Enlargement of optic nerves or optic chiasm (diameter over 3.9 mm). On T2-weighted scans: core with low signal surrounded by a higher intensity ring. On T1-weighted scans: isointense signal, showing enhancement after gadolinium administration.

**Table 4:** Recent clinical studies with NF1.

Study	Study Design	Patient Population	n	Age Range	Primary Endpoint	Response Rate	Key Adverse Events	Reference
SPRINT Stratum 1	Phase II, single-arm, open-label	Pediatric NF1 with inoperable PN	50	3.5–17.4 years median 10.2	≥20% reduction in PN volume by MRI at 12 months	35/50 (70%) partial response, 28/50 (56%)durable response	Vomiting (42%), rash (40%), abdominal pain (38%), diarrhea (36%)	Gross et al., 2020 [20]
SPRINT Stratum 2	Phase II, single-arm, open-label	Pediatric NF1 with asymptomatic PN at risk	25	4.5–18.1 years median 12.3	≥20% reduction in PN volume by MRI	PN shrinkage in 18/25 (72%) subjects	Similar to Stratum 1; no grade 4 events	Gross et al., 2022 [21]
Pediatric Phase I	Phase I, dose- escalation	Pediatric patients with recurrent or refractory solid tumors	24	3.0–18.5 years median 10.8	Maximum tolerated dose (25 mg/m^2^ BID)	Partial response of 17 of the 24 children (71%), decreases from baseline in neurofibroma volume in 12 of 18 mice (67%), progression (tumor volume increase from baseline of ≥20%) has not been observed.	Rash (71%), vomiting (45%), diarrhea (42%)	Dombi et al., 2016 [19]

**Abbreviations:** NF1 = neurofibromatosis type 1; PN = plexiform neurofibroma; MRI = magnetic resonance imaging; BID = twice daily.

## Data Availability

Not applicable.
